# Application of artificial intelligence in the diagnosis of multiple primary lung cancer

**DOI:** 10.1111/1759-7714.13185

**Published:** 2019-09-17

**Authors:** Xin Li, Bin Hu, Hui Li, Bin You

**Affiliations:** ^1^ Department of Thoracic Surgery Beijing Chao‐Yang Hospital Affiliated Capital Medical University Beijing China

**Keywords:** 3D volume, AI, follow‐up, multiple primary lung cancer

## Abstract

Artificial intelligence (AI) based on deep learning, convolutional neural networks and big data has been increasingly effective in the diagnosis and treatment of multiple primary pulmonary nodules. In comparison to previous imaging systems, AI measures more objective parameters such as three‐dimensional (3D) volume, probability of malignant nodules, and possible pathological patterns, making the access to the properties of nodules more objective. In our retrospective study, a total of 53 patients with synchronous and metachronous multiple pulmonary nodules were enrolled of which 33 patients were confirmed by pathological tests to have primary binodules, and nine to have primary trinodules. A total of 15 patients had only one focus removed. The statistical results showed that the agreement in the AI diagnosis and postoperative pathological tests was 88.8% in identifying benign or malignant lesions. In addition, the probability of malignancy of benign lesions, preinvasive lesions (AAH, AIS) and invasive lesions (MIA, IA) was totally different (49.40±38.41% vs 80.22±13.55% vs 88.17±17.31%). The purpose of our study was to provide references for the future application of AI in the diagnosis and follow‐up of multiple pulmonary nodules. AI may represent a relevant diagnostic aid that shows more accurate and objective results in the diagnosis of multiple pulmonary nodules, reducing the time required for interpretation of results by directly displaying visual information to doctors and patients and together with the clinical conditions of MPLC patients, offering plans for follow‐up and treatment that may be more beneficial and reasonable for patients. Despite the great application potential in pneumosurgery, further research is needed to verify the accuracy and range of the application of AI.

## Introduction

In 2018, reports from the National Central Cancer Registry of China (NCCRC) showed that lung cancer is the malignant tumor with the highest incidence of 57.13/10^5^.^1^ The incidence of pulmonary adenocarcinoma is increasing, and this disease accounts for over 40% of the cases of non‐small cell lung cancer by preliminary diagnosis.^2^ The rate of detectable early lung cancer has improved with the application of high‐resolution computed tomography (HRCT). The incidence of multiple primary lung cancer is 0.2%–20%.^3^ The National Comprehensive Cancer Network (NCCN) and the American College of Chest Physicians (ACCP) issued in 2017 and 2013, respectively, recommendations for follow‐up and treatment of ground‐glass nodules (GGNs), where the evaluation patterns were primarily based on the diameter of the nodule, proportion of solid lesion, risk level and changes found at follow‐up, and the radiologic characteristics of GGNs limited to the cross‐sectional diameter of nodules. It has been suggested in the literature that nodule three‐dimensional (3D) volume and longest 3D diameter may show better actual characteristics of growth of irregular nodules, improving both the accuracy and reproducibility of the diagnosis of pulmonary nodules.^4^, ^5^ In recent years, medical artificial intelligence (AI) has been developed based on computer science, information science and big data using deep learning and convolutional neural network (CNN). Given the rising incidence of lung cancer, the application of AI for the screening and diagnosis of pulmonary nodules is an interesting research topic. Studies have indicated that the sensitivity and specificity of AI in reading chest CT images of Stage IA lung cancer was 96.40% and 95.60%, respectively. In this study, we report the results of a retrospective study comparing pathological findings and AI diagnosis of patients with multiple pulmonary nodules. Our investigation aimed to survey the agreement of AI diagnosis and pathological diagnosis in the hope of providing evidence to any future application of AI in follow‐up or surgical strategies for multiple pulmonary nodules.

## Methods

### Patients

A total of 53 patients (12 males, 41 females; ages: 27–82 years old, average 60.5 ± 9.09 years) with multiple lesions, who underwent operative treatments from March 2017 to December 2018 in our department, were enrolled to receive HRCT before a surgery which included, according to their lung functions and lesion sites, video‐assisted thoracoscopic surgery (VATS) for pulmonary lobe resection, segmental resection of lung or wedge resection of lung. For those patients who had multiple ipsilateral lesions, combined resection was performed and regular postoperative follow‐up was conducted to prevent further operations. Meanwhile, systemic lymph node dissection was applied, and the resected tissues were sent for pathological examination.

### Instruments and methods

A Siemens spiral CT scanner was used to scan the patient who, in the supine position, took a deep breath and then held their breath from the apex pulmonis to the basis pulmonis. Conditions of scanning were: voltage 120 kV, current 130 mA, layer thickness 0.67 mm, matrix 512 × 512. Original data were transferred to the GE workstation and successive images saved in dicom format. The AI system called A'Eyes‐Care, provided by Diannei, was used to view the dicom files and calculate 3D volumes in addition to parameter estimation.

### Pathological diagnosis

Specimens of lung tissues and lymph nodes were fixed, embedded and sliced for HE and immunohistochemical staining before being analyzed by a senior pathologist. The identification of pulmonary adenocarcinoma was in accordance with the 2011 IASLC/ATS/ERS International Multidisciplinary Classification and Diagnosis of Pulmonary Adenocarcinoma: (i) Atypical adenomatous hyperplasia: intrapulmonary lesions <5 mm, local type II alveolar cell and/or Clara cell proliferative lesions. (ii) Adenocarcinoma in situ: localized adenocarcinoma <3 cm; cancer cells grow by covering walls; no virtual, vascular or pleural infiltration; no papilla or papilla structures; accumulation of cancer cells is not seen in the alveolar space. (iii) Minimally invasive adenocarcinoma: localized; ≤3 cm; cancer cells grow by mainly covering walls, and the maximum diameter of interstitial infiltration in any field was ≤0.5 cm. (iv) Invasive adenocarcinoma: the maximum diameter of interstitial infiltration in at least one field was ≥0.5 cm.

### Artificial intelligence aids in diagnosis

The AI diagnosis system used in the study was supported and maintained by Diannei Technology Co. Ltd (Shanghai, China). The core diagnosis component of the AI system was 3D DenseSharp Network,^6^ a state‐of‐the‐art multitask learning deep neural network based on 3D DenseNets,^7^ with classification and segmentation heads for diagnosing and segmenting lung nodules. In the primary study on cross‐modal pathological invasiveness prediction from CT scans, the software developer set up a single‐cohort data set, which involved 651 patients from Huadong Hospital affiliated to Fudan University, Shanghai, China.^6^ Each sample in this data set consisted of the CT scan, the corresponding pathology‐validated invasiveness label (AAH, AIS, MIA, or IA), and expert‐annotated lesion segmentation. Based on this data set, the 3D DenseSharp Network was developed to train and predict the invasiveness labels and lesion segmentation, with a superior performance over radiologists on this task. The AI system used in this study, developed with the 3D deep learning technology,^6^, ^7^ took advantage of a multicohort data set, with 10x more patients than their primary study.^6^ Using this AI system, dicom files were analyzed to screen out intrapulmonary subsolid nodules and their three‐dimensional (3D) volumes, radial lines, probability of nodules and malignancy, probable pathological patterns and other parameters. (i) 3D volume was calculated by AI through pixel‐level scanning and automatic segmentation of nodules. (ii) Probability of nodular and malignant fitting between the detected characteristics of the nodules and pathological features established from deep learning was performed. A higher probability meant a greater likelihood of a nodular or malignant lesion. In this study, the threshold of output of nodular probability was 0.3, which controlled the output quantity of the identified nodules. (iii) Probable patterns: AAH, AIS, MIA, IA, or unknown. The infiltration was determined based on the fitting by the AI of detected nodules and characteristics of nodules with various pathological patterns.

### Statistics

IBM SPSS25.0 statistical analysis software was used. All measured data were expressed as the mean ± standard deviation (mean ± SD). In terms of means within groups, the *t*‐test with two independent samples was applied if the data were subject to a normal distribution; otherwise, the rank‐sum test was used. Classified variables were analyzed with the chi‐square test. *P* < 0.05 was considered to be significantly different. Cutoff points from the ROC curve were used to evaluate the efficiency of parameters, such as probability of malignancy, in indicating the possible lesions.

## Results

The 53 patients that underwent surgical treatment in our department during the period March 2017 to December 2018 included 33 patients with binodules, nine with trinodules and 15 who had only one focus removed. Simultaneous surgery was conducted for 48 patients whose lesions were all ipsilateral, and surgery by stages was conducted for five patients, of which three had ipsilateral and two bilateral lesions. The statistical data in this paper were acquired from the 108 resected lesions of the 53 patients. Additionally, it was found that females accounted for 77.3% of the occurrence of multiple primary lung cancer (MPLC), and only 4.8% of the cases in our study were smokers.

### AI and pathological findings

Tables 1 and 2 showed the statistical analysis of pathological diagnosis and AI diagnosis. The probabilities of benign nodules, malignant preinvasive lesions (AAH, AIS) and malignant invasive lesions (MIA, IA) were 49.40 ± 38.41%, 80.22 ± 13.55% and 88.17 ± 17.31%, respectively, and the agreement rate of malignancy was 69.2%, 88.2% and 90.2%. The overall agreement rate of malignancy was 88.8%. However, in this retrospective study, the cases involved were all considered to be potentially malignant by clinicians, and the proportion of benign lesions by pathological diagnosis was 22 patients (20.2%), which may have caused selection bias. In addition, only six patients had AAH and MIA (5.5%). This observation may be associated with the diagnostic preference of the pathologists.

**Table 1:** Comparison of pathological and AI diagnosis

AI pathological	Total	Benign /unknown	AAH	AIS	MIA	IA	Unidentified	Three dimensional (3D) volume	Probability of nodular	Probability of malignant	Pathological agreement completely	Agreement of malignancy
Benign	22	9	1	1	1	1	9	504.73 ± 1182.42	73.11 ± 28.49	49.40 ± 38.41	9/13	9/13
AAH	5	1	0	2	1	0	1	/	/	/	1/4	3/4
AIS	31	3	2	15	4	5	1	1501.03 ± 2459.52	87.55 ± 16.01	79.95 ± 13.40	15/30	27/30
MIA	1	0	0	1	0	0	0	/	/	/	/	/
IA	47	4	1	13	0	23	6	3831.84 ± 4335.74	83.26 ± 17.29	88.10 ± 17.16	23/41	37/41
SCC	1	/	/	/	/	1	/	1716	94.66	98.91	0/1	1/1
Metastatic carcinoma	2	/	/	/	/	/	2	/	/	/	/	/

**Table 2:** Comparison of pre‐ and invasive lesions

	Preinvasive (AAH, AIS)	Invasive (MIA, IA)	
Three‐dimensional (3D) volume	1339.00 ± 2310.84	3670.96 ± 4291.21	*P* = 0.000
Probability of nodules	84.20 ± 20.05	83.81 ± 17.04	*P* = 0.613
Probability of malignancy	80.22 ± 13.55	88.17 ± 17.31	*P* = 0.001
Complete pathological agreement	15/34	23/41	*P* = 0.302
Agreement of malignancy	30/34	37/41	*P* = 0.510

Probability of nodules and probability of malignancy were subjected to the Mann‐Whitney U rank‐sum test.

Agreements with pathological patterns and accuracy in diagnosis of malignancy were subjected to the chi‐square test.

### Comparison of benign and malignant lesions

The AI system detected 89 (82.4%) nodules in total, and 19 (17.6%) nodular lesions were overlooked. The agreement in benign lesions was not perfect due to the nine cases of solid lesions being overlooked during detection by AI and the small sample size. This result might be explained by the fact that clinicians normally do not choose surgical excision in cases of typical benign lesions. The detectable rate and diagnosis of benign nodules, especially solid benign nodules, by AI will be further surveyed with larger samples. On the other hand, the accuracy in the diagnosis of 89 malignant subsolid lesions by AI was 88.8%, which is more credible, and the area under the ROC curve was 0.766. Further, the probability of malignancy was 75.04%, which was the cutoff point in determining the malignancy. The sensitivity and false positive rates were 75.0% and 30.8%, respectively. Of the 37 patients for whom the AI diagnosis was not in line with the pathological diagnosis, eight malignant nodules were identified by AI as unknown or benign. AI showed incapacity of recognition for solid mass lesions and central tumors of ≥2 cm. The 19 lesions not identified were solid mass lesions suggesting the irreplaceable importance of the reading work done by radiologists.

### Preinvasive and invasive lesions

**Figure 1:**
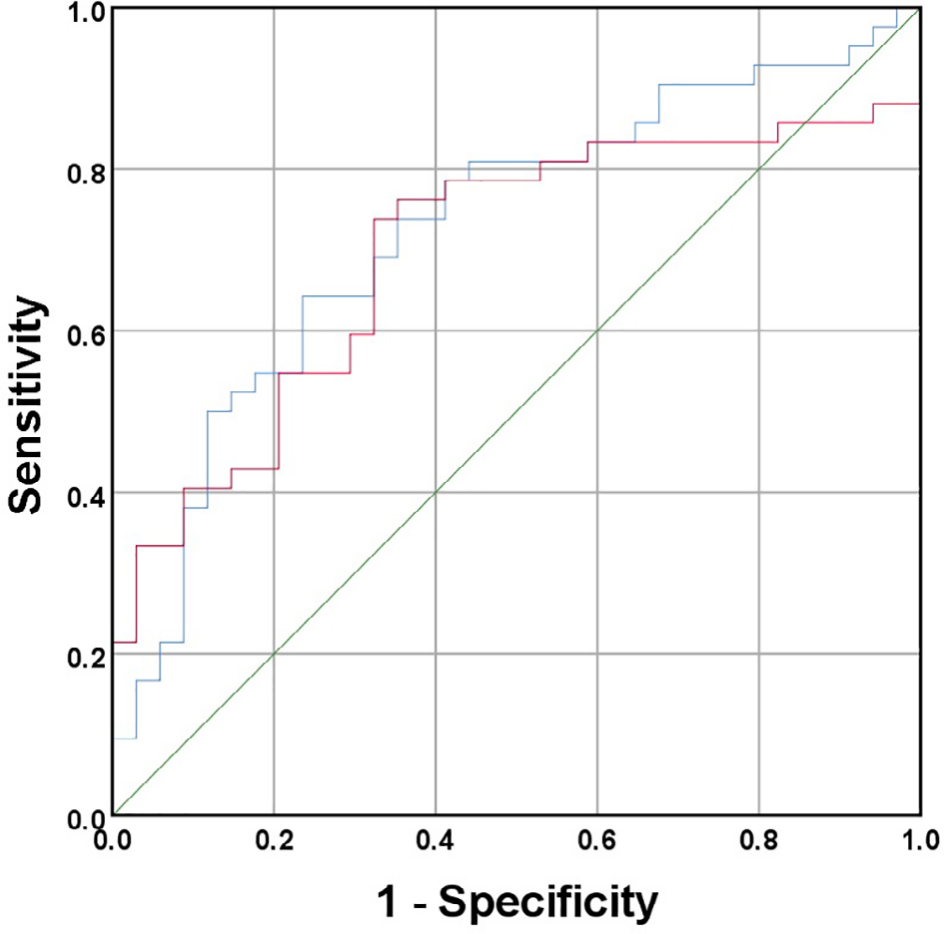
Three‐dimensional (3D) volume and probability of malignancy in determining the invasion or not. Source of curve (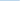) 3D volume, (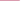) probability of malignancy and (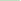) reference line.

Table 2 showed statistical significance in the 3D volume and probability of malignancy for both preinvasive and invasive lesions (*P* < 0.01). Pathological agreement and agreement of malignancy, however, were not statistically significant (*P* > 0.05). The invasive ROC curve indicated that the areas under the curve for 3D volume and probability of malignancy were 0.726 and 0.696, respectively (Fig 1). The cutoff points in determining the invasiveness were 1089.5 mm^3^ and 88.24%. These cutoff points may be references for the future selection of operation modes.

### Patients receiving imaging follow‐up examination

**Figure 2:**
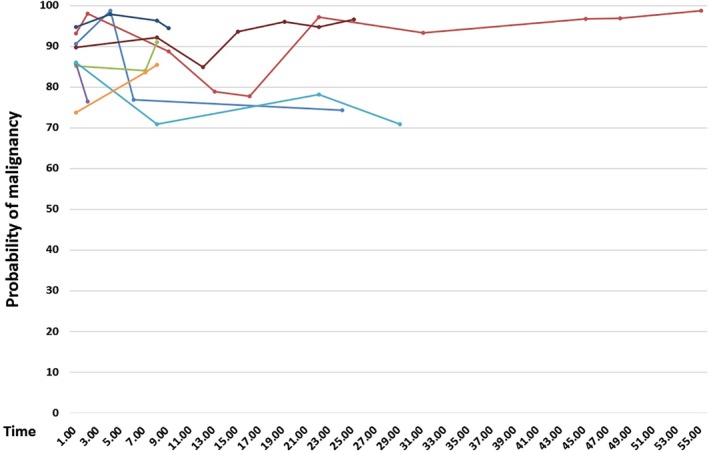
Trend of probability of malignancy in cases receiving imaging follow‐up examination.

**Figure 3:**
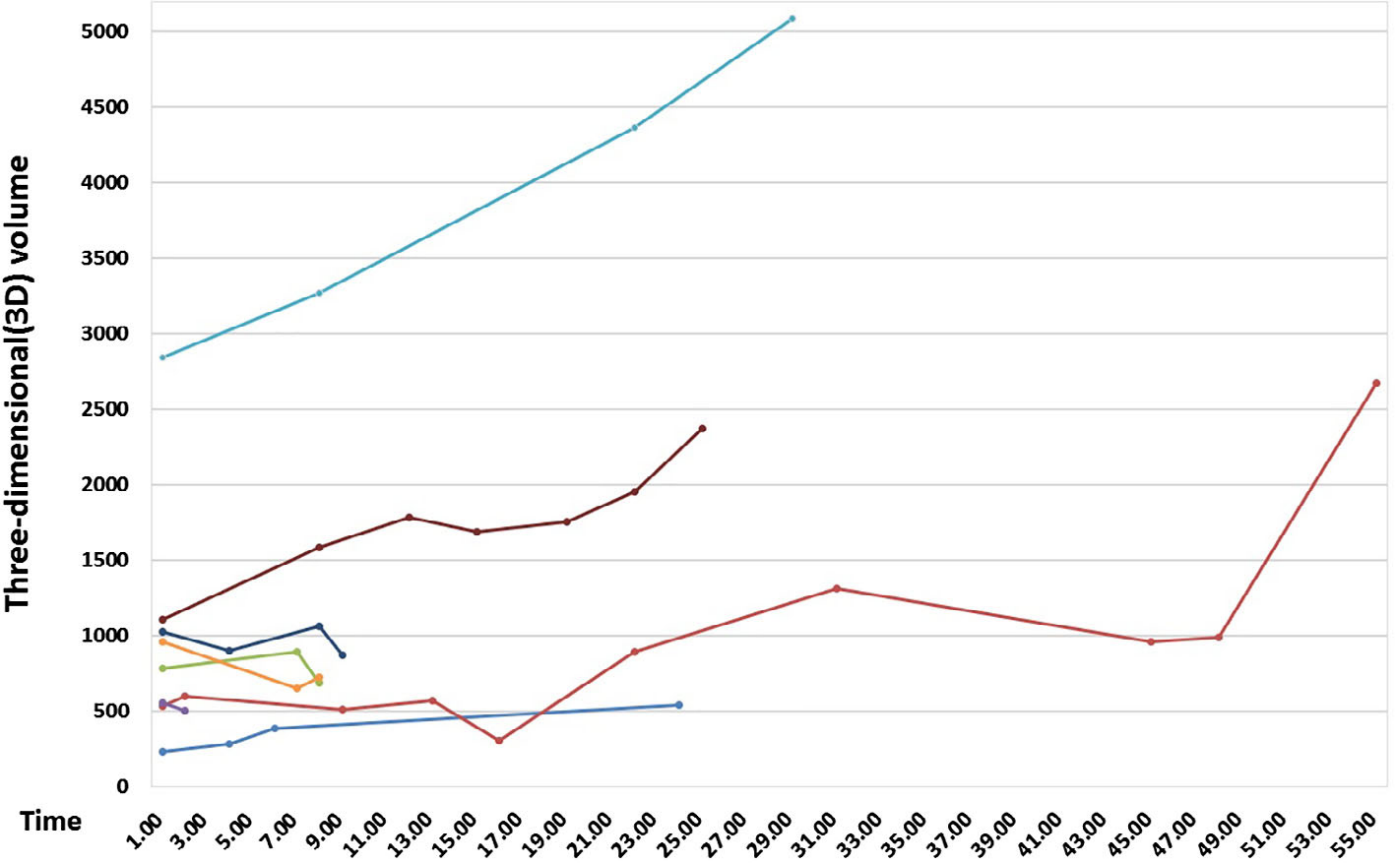
Trend of three‐dimensional (3D) volume in cases receiving imaging follow‐up examination.

In Figures 2 and 3, the multi‐temporal‐point AI analysis of eight cases of adenocarcinoma confirmed by postoperative pathology in seven patients, who were followed up for a long time before surgery, showed that the probabilities of malignancy did not increase over time but fluctuated stably in the interval of 70%–99%, which is in good agreement with the cutoff point of 75.04%. In patients undergoing follow‐up of over 12 months, their 3D volumes of lesions were significantly increased, while in patients with follow‐up of less than one year, the fluctuations in 3D volumes of lesions were unclear. AI may provide some indications for patients whose operational procedures may be confirmed only after follow‐up. However, currently, the information obtained therein cannot be used to generate the perfect follow‐up intervals and times and further research is warranted.

### Lesions not identified by AI

Of the 19 lesions not detected by the AI system, 10 were solid nodules, one included multiple nodules in both sides combined with miliary nodules, and eight were unidentified. These accounted for 17.59% among the surgically removed lesions. In the review of CT, it was found that such unrecognized lesions were mostly solid lesions or lesions close to the mediastinum or diaphragm and adjacent to the surrounding high‐density tissues. There were also many other cases with interstitial or chronic inflammatory changes in the lungs.

## Discussion

In the treatment of lung cancer, early detection and diagnosis have always been critical. A sensitive and accurate diagnostic method may represent, with the increasing incidence of lung cancer, not only an important tool used by clinicians but also the key to improving survival rates. Recently, AI has been developed on the basis of computer science, information science and big data, leading to the development of popular AI systems for pulmonary subsolid nodules. Given the increasing number of multiple pulmonary nodules observed in the clinic, we present a retrospective study designed to evaluate the application of AI in multiple pulmonary nodules with respect to pathological diagnosis, probability of malignancy, 3D parameters and long‐term follow‐up findings.

### Clinical significance of three‐dimensional parameters and probability of malignancy

Numerous investigations have been implemented in recent years by clinicians and imaging doctors regarding the diagnosis and treatment of pulmonary nodules to improve the early screening and diagnosis of lung cancer and the treatment based on well‐documented clinical guidelines, to avoid overtreatment due to subjective judgments. The Fleischner Association and NCCN released in 2017 and 2016, respectively, the latest clinical guidelines in which the former evaluated lung nodules using 3D volume measurements.^5^ In addition, studies have shown that 3D volumetric measurements provide good accuracy and reproducibility.^8^ Since then, a variety of predictive models based on 3D reconstruction have been designed and applied by researchers, which have measured the mass, volume and solid proportion for regression analyses and drawn conclusions based on 3D volume or solid proportion with the contribution of computer‐aided calculations. Mehta *et al*. applied a nodular volume of 0.5 cm^3^ as the cutoff point in the diagnosis of benign or malignant pulmonary nodules.^9^ Yu considered that 3D measurements were more accurate in identifying invasive adenocarcinoma and used a solid proportion of 74% and proportion of solid mass of 0.7 cm^3^ as the cutoff point in identifying benign or malignant pulmonary nodules.^10^

Table 2 displays the statistical significance of the 3D volume of nodules in the evaluation of invasive lesions (*P* < 0.01). The 3D volume of 1089.5 mm^3^ and the probability of malignancy of 88.24% may be the cutoff point of invasion or noninvasion. These cutoff parameters, in such an era of anatomical segmental resection of the lung, may become one of the objective references for surgeons in determining the appropriate surgical procedures for those with highly suspected invasive lesions to avoid an invalid operation period; this approach would be unnecessary if a lobectomy of the lung was initially selected, but when there is a need for remedial lobectomy of the lung after an anatomical segmental resection, this approach would reduce the anesthesia period and improve the long‐term outcome.

### AI and pathological diagnosis

In a comparison of AI diagnosis and postoperative pathological results, Tables 1 and 2 showed that the accuracy of AI in identifying benign or malignant lesions was 88.8%, whereas the probability of malignancy in benign nodules, preinvasive lesions (AAH, AIS) and invasive lesions (MIA, IA) were 49.40 ± 38.41%, 80.22 ± 13.55% and 88.17 ± 17.31%, respectively, and the optimum cutoff point of malignancy was 75.04%. These calculations were based on the evaluation of nodules and the pixel‐level fitting by AI, which objectively reflects the fit of the detected lesions to various pathological patterns in the database. The higher agreement in malignant diagnosis may also be attributed to the fact that the patients included in the analysis have all been suspected by surgeons as having a greater likelihood for malignant nodules, and this fact may cause selection bias. In this study, the optimum cutoff point of malignancy was confirmed as 75.04%, which certainly will require further confirmation using a larger sample size and updated AI system. Although different AI systems may give different optimal cutoff points, favorable objectivity and reproducibility of AI are worthy of consideration by clinicians and serve as vital diagnostic references.

### Follow‐up with CT and AI

Among the patients with multiple nodules, a large proportion presented with bilateral multiple nodules requiring prompt phased surgeries or several rounds of follow‐up, during which the clinicians had to compare the multiple CT imaging and evaluate the operation opportunities and indicators, such as diameter, shape, and proportion of the solid mass of the nodule. Such laborious comparison of CT images in order to grasp the changes in multiple nodules is very time‐consuming and cannot generate the visual findings that may be easily displayed by objective data; however, in AI systems, the 3D volume and probability of malignancy of the nodules directly and visually mirror the patterns of changes and provide better reproducibility. Figures 2 and 3 show the CT scans of patients with multiple nodules. The probabilities of malignancy in lesions being surveyed during a follow‐up period ranging from nine months to four years fluctuated between 70%–99%, and most of these lesions showed great increase in 3D volumes. The probability of malignancy showed more stability for the evaluation of lesions and did not considerably change over time, while the 3D volume of lesions may be a more sensitive reference for the assessment of nodule progression to assist physicians in determining the best treatment.

### AI diagnosis and surgery

At present, the most common application of AI in the clinic is detecting and displaying lung nodules and providing reference materials for clinicians. In the case of patients with multiple bilateral or unilateral nodules, the clinicians may determine the priority of surgical resection in such conditions based on the malignancy, size and shape of the lesions. At this point, the diagnosis by AI may helpfully provide objective materials that can contribute to the selection of resection, segmentectomy or combined resection of the lung. However, AI systems currently simply analyze CT sequence files and cannot integrate the clinical information and medical history for a comprehensive analysis, which is just an irreplaceable tool for clinicians.

### Development of AI

Continuous expansion of AI system databases will improve the accuracy and recognition range. This expansion will greatly shorten analysis time and be a time‐saving tool for clinicians. In patients with multiple nodules, AI provides visual comparison and follow‐up materials which avoid the complicated human‐made contrast and provide objective and quantitative evidence for the selection of surgical procedures. In addition, AI systems, with the support of high speed networks, may in the future be a platform for remote consultations, where the established cloud diagnosis system serves patients in community hospitals, or hospitals in remote regions who require prompt and efficient medical treatment and may be extended to many other applications upon database improvement and continuous exploration. The widescale development and application of AI requires us to maintain a conservative attitude, while actively verifying the accuracy and scope of the applications in a cautious manner. In future, AI should be developed with the function of clinical information integration and improved algorithmic models coming from an expanded database, and, if possible, a reliable and efficient platform for diagnosis and follow‐up.

## Disclosure

The authors declare that they have no conflicts of interest and that there are no commercial or associative interest that represents a conflict of interest in connection with the work submitted.
